# 3-(2,2-Dioxo-3,4-di­hydro­benzo[*e*][1,2,3]oxa­thiazin-4-yl)-3-fluoro-1-phenyl­indolin-2-one

**DOI:** 10.1107/S2414314620010287

**Published:** 2020-07-31

**Authors:** Mei-Fang Wu, Ling-Yan Chen, Ya Li

**Affiliations:** a College of Chemistry and Chemical Engineering, Shanghai University of Engineering Science, 333 Longteng Road, Shanghai 201620, People’s Republic of China; Goethe-Universität Frankfurt, Germany

**Keywords:** crystal structure, fluorine, sulfamidate

## Abstract

The title compound, C_21_H_15_FN_2_O_4_S, contains two chiral carbon centres, but crystal symmetry generates a racemic mixture. The crystal structure features N—H⋯O hydrogen bonding. The sulfonate group is disordered with an occupancy ratio of 0.933 (4):0.067 (4).

## Abstract 2

The title compound contains two chiral carbon centres. It has monoclinic (*P*2_1_/*c*) symmetry. The structure displays N—H⋯O hydrogen bonding.

## Graphical abstract

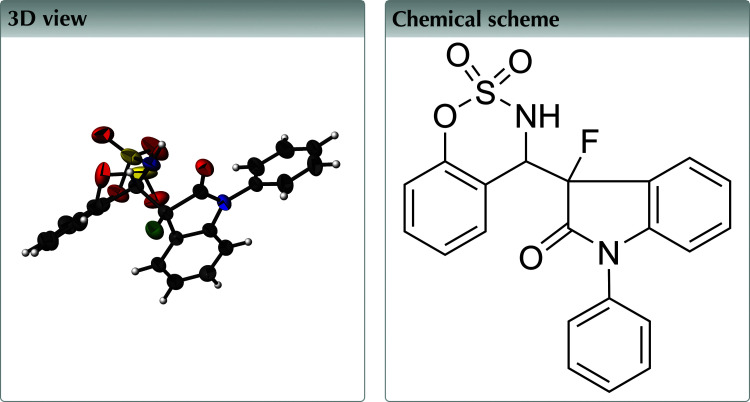

## Structure description

**Figure 1:**
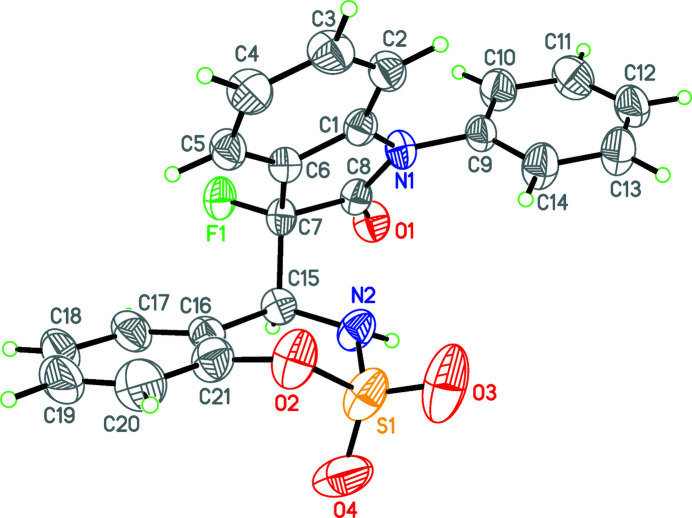
The mol­ecular structure of the title compound. Displacement ellipsoids are drawn at the 50% probability level. Only the major disorder component is shown

The incorporation of one or more fluorine atoms into an organic mol­ecule can result in improved thermal/metabolic stability, bioactivity and lipophilicity (Purser *et al.*, 2008 ▸). In this context, the β-fluoro­amine motif is an important structural feature and has been found in a number of drug candidates (Zhao *et al.*, 2019 ▸). Consequently, the synthesis of chiral mol­ecules with a fluorinated carbon center has attracted recent attention (Shang *et al.*, 2015 ▸; Chen *et al.*, 2017 ▸; Paladhi *et al.*, 2017 ▸; Zheng *et al.*, 2018 ▸). As part of our work in this area, we now describe the synthesis and structure of the title compound (Fig. 1 ▸).

**Figure 2:**
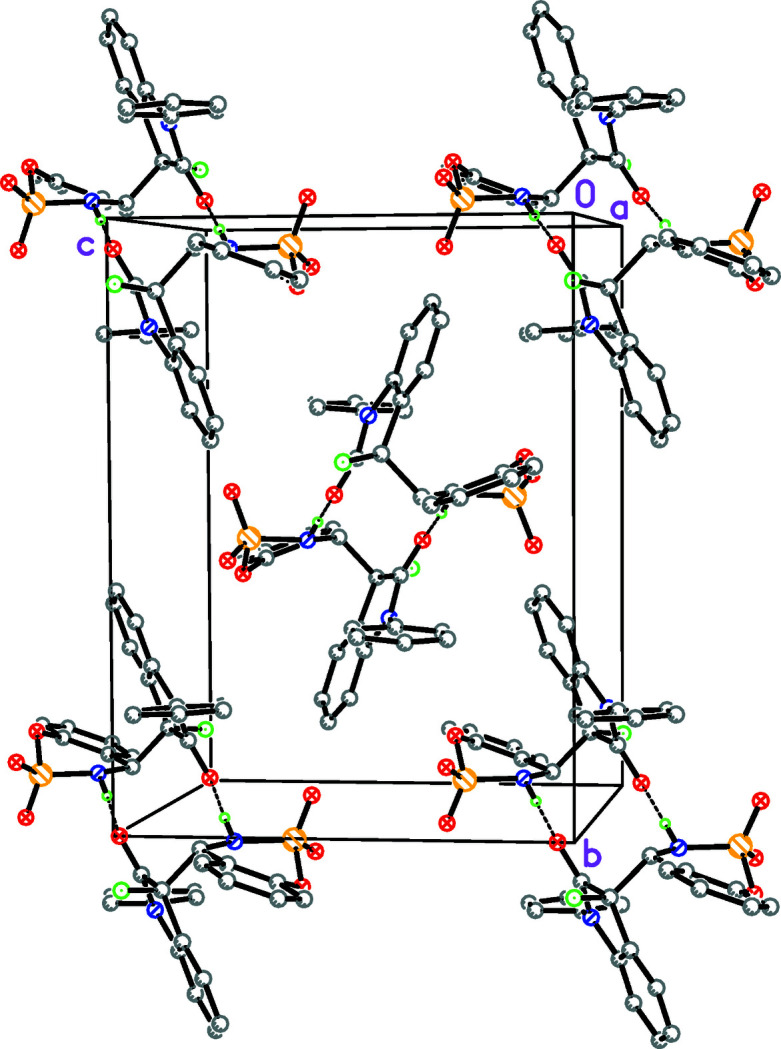
A view of the packing diagram showing the centrosymmetric dimers with N—H⋯O hydrogen bonds as dashed lines.

The geometric parameters do not show any unusual features. In the crystal, mol­ecules are connected by pairwise N—H⋯O hydrogen bonds (Table 1 ▸, Fig. 2 ▸) to generate centrosymmetric *R* ^2^_2_(12) loops.

## Synthesis and crystallization

Under an N_2_ atmosphere, a 10 mL reaction tube was charged with 3-fluoro-1-phenyl­indolin-2-one (0.24 mmol), catalyst 4-[(*S*)-(benz­yloxy)(1*S*,2*R*,4*S*,5*R*)-5-vinyl­quinuclidin-2-yl]meth­yl)quinolin-6-ol (12.0 mg, 0.03 mmol) and dried CHCl_3_ (2.0 ml). The reaction mixture was cooled to 0°C, followed by the addition of benzo[*e*][1,2,3]oxa­thia­zine 2,2-dioxide (0.2 mmol). The reaction mixture was stirred at 0°C until the complete conversion of benzo[*e*][1,2,3]oxa­thiazine 2,2-dioxide, and was then purified by flash chromatography to give the desired product (80.4 mg, 98%). Crystals were grown from petroleum ether/ethyl acetate solution. Data: [α]22^D^= −32.9 (*c* = 0.54, CHCl_3_); m.p. 210.2–211.3°C. ^1^H NMR (500 MHz, DMSO-*d*_6_) *δ* 9.37 (*s*, 1H), 7.81 (*d*, *J* = 7.5 Hz, 1H), 7.65 (*d*, *J* = 7.7 Hz, 1H), 7.62 (*d*, *J* = 7.2 Hz, 1H), 7.59 (*d*, *J* = 7.8 Hz, 1H), 7.53 (*t*, *J* = 7.4 Hz, 1H), 7.46 (*t*, *J* = 6.7 Hz, 3H), 7.36 (*t*, *J* = 7.7 Hz, 1H), 7.24 (*d*, *J* = 8.2 Hz, 1H), 6.94 (*t*, *J* = 7.6 Hz, 1H), 6.76 (*d*, *J* = 7.9 Hz, 1H), 6.51 (*d*, *J* = 7.4 Hz, 1H), 5.52 (*d*, *J* = 12.6 Hz, 1H). ^19^F NMR (471 MHz, DMSO-*d*_6_) *δ* −150.20 (*s*). ^13^C NMR (126 MHz, DMSO-*d*_6_) *δ* 169.95 (*d*, *J* = 20.0 Hz), 151.80 (*s*), 145.74 (*d*, *J* = 6.2 Hz), 133.63 (*s*), 132.67 (*d*, *J* = 3.2 Hz), 131.46 (*s*), 130.38 (*s*), 129.20 (*s*), 129.00 (*d*, *J* = 7.7 Hz), 126.93 (*s*), 126.21 (*d*, *J* = 4.5 Hz), 123.76 (*d*, *J* = 2.9 Hz), 122.17 (*s*), 122.03 (*s*), 119.37 (*s*), 118.12 (*d*, *J* = 1.7 Hz), 110.44 (*s*), 93.67 (*d*, *J* = 190.8 Hz), 58.82 (*d*, *J* = 35.1 Hz).

## Refinement

Crystal data, data collection and structure refinement details are summarized in Table 2 ▸. The disordered sulfonate group was treated using a PART command in the refinement. The occupancy factors were restrained to sum to unity. The refined occupancy ratio is 0.933 (4):0.067 (4). Atomic displacement parameters of S1 and O3 were restrained using a DELU command.

## Supplementary Material

Crystal structure: contains datablock(s) I. DOI: 10.1107/S2414314620010287/bt4093sup1.cif

Structure factors: contains datablock(s) I. DOI: 10.1107/S2414314620010287/bt4093Isup3.hkl

Supporting information file. DOI: 10.1107/S2414314620010287/bt4093Isup3.cml

CCDC reference: 1971616

Additional supporting information:  crystallographic information; 3D view; checkCIF report

## Figures and Tables

**Table 1:** Hydrogen-bond geometry (Å, °)

*D*—H⋯*A*	*D*—H	H⋯*A*	*D*⋯*A*	*D*—H⋯*A*
N2—H12⋯O1^i^	0.89 (3)	1.99 (3)	2.868 (2)	166 (2)

Symmetry code: (i) 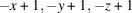 .

**Table 2:** Experimental details

Crystal data	
Chemical formula	C_21_H_15_FN_2_O_4_S
*M*_r_	410.41
Crystal system, space group	Monoclinic, *P*2_1_/*c*
Temperature (K)	292
*a*, *b*, *c* (Å)	9.8726 (4), 15.8054 (7), 11.8345 (4)
β (°)	94.679 (1)
*V* (Å^3^)	1840.51 (13)
*Z*	4
Radiation type	Mo *K*α
μ (mm^−1^)	0.22
Crystal size (mm)	0.18 × 0.15 × 0.10

Data collection	
Diffractometer	Bruker APEXII CCD
Absorption correction	Multi-scan (*SADABS*; Bruker, 2014 ▸)
*T*_min_, *T*_max_	0.671, 0.746
No. of measured, independent and observed [*I* > 2σ(*I*)] reflections	9057, 3596, 2580
*R*_int_	0.030
(sin θ/λ)_max_ (Å^−1^)	0.617

Refinement	
*R*[*F* ^2^ > 2σ(*F* ^2^)], *wR*(*F* ^2^), *S*	0.044, 0.110, 1.08
No. of reflections	3596
No. of parameters	351
No. of restraints	1
H-atom treatment	All H-atom parameters refined
Δρ_max_, Δρ_min_ (e Å^−3^)	0.21, −0.23

Computer programs: *APEX2* and *SAINT* (Bruker, 2014 ▸), *SHELXS97* and *SHELXTL* (Sheldrick 2008 ▸) and *SHELXL2014/7* (Sheldrick, 2015 ▸).

## full crystallographic data

### Crystal data

**Table:**

C_21_H_15_FN_2_O_4_S	*F*(000) = 848
*M**_r_* = 410.41	*D*_x_ = 1.481 Mg m^−^^3^
Monoclinic, *P*2_1_/*c*	Mo *K*α radiation, λ = 0.71073 Å
*a* = 9.8726 (4) Å	Cell parameters from 3064 reflections
*b* = 15.8054 (7) Å	θ = 4.9–54.4°
*c* = 11.8345 (4) Å	µ = 0.22 mm^−^^1^
β = 94.679 (1)°	*T* = 292 K
*V* = 1840.51 (13) Å^3^	Prismatic, colorless
*Z* = 4	0.18 × 0.15 × 0.10 mm

### Data collection

**Table:**

Bruker APEXII CCD diffractometer	2580 reflections with *I* > 2σ(*I*)
φ and ω scans	*R*_int_ = 0.030
Absorption correction: multi-scan (SADABS; Bruker, 2014)	θ_max_ = 26.0°, θ_min_ = 2.9°
*T*_min_ = 0.671, *T*_max_ = 0.746	*h* = −12→12
9057 measured reflections	*k* = −19→16
3596 independent reflections	*l* = −13→14

### Refinement

**Table:**

Refinement on *F*^2^	Hydrogen site location: difference Fourier map
Least-squares matrix: full	All H-atom parameters refined
*R*[*F*^2^ > 2σ(*F*^2^)] = 0.044	*w* = 1/[σ^2^(*F*_o_^2^) + (0.0419*P*)^2^ + 0.4204*P*] where *P* = (*F*_o_^2^ + 2*F*_c_^2^)/3
*wR*(*F*^2^) = 0.110	(Δ/σ)_max_ < 0.001
*S* = 1.08	Δρ_max_ = 0.21 e Å^−^^3^
3596 reflections	Δρ_min_ = −0.23 e Å^−^^3^
351 parameters	Extinction correction: SHELXL-2014/7 (Sheldrick 2014, Fc^*^=kFc[1+0.001xFc^2^λ^3^/sin(2θ)]^-1/4^
1 restraint	Extinction coefficient: 0.022 (2)

### Special details

**Table:**

Geometry. All esds (except the esd in the dihedral angle between two l.s. planes) are estimated using the full covariance matrix. The cell esds are taken into account individually in the estimation of esds in distances, angles and torsion angles; correlations between esds in cell parameters are only used when they are defined by crystal symmetry. An approximate (isotropic) treatment of cell esds is used for estimating esds involving l.s. planes.
Refinement. The position of H(2) atom was found from the diagram of differential Fourier synthesis.

### Fractional atomic coordinates and isotropic or equivalent isotropic displacement parameters (Å^2^)

**Table:**

	*x*	*y*	*z*	*U*_iso_*/*U*_eq_	Occ. (<1)
S1	0.37546 (9)	0.45878 (11)	0.17560 (7)	0.0574 (4)	0.933 (4)
O2	0.2617 (2)	0.39173 (17)	0.13707 (18)	0.0585 (7)	0.933 (4)
O4	0.3356 (2)	0.53877 (14)	0.12761 (16)	0.0804 (8)	0.933 (4)
O3	0.50142 (19)	0.42592 (16)	0.15129 (17)	0.0930 (8)	
S1'	0.3792 (17)	0.4087 (16)	0.1887 (14)	0.079 (5)	0.067 (4)
O2'	0.251 (4)	0.452 (3)	0.118 (3)	0.075 (11)	0.067 (4)
O4'	0.373 (3)	0.3271 (18)	0.191 (2)	0.076 (11)	0.067 (4)
F1	0.13032 (11)	0.39571 (8)	0.51531 (10)	0.0450 (4)	
O1	0.40253 (15)	0.45785 (9)	0.58419 (12)	0.0448 (4)	
N1	0.45048 (16)	0.32572 (11)	0.51448 (13)	0.0363 (4)	
N2	0.36500 (19)	0.45557 (12)	0.31047 (15)	0.0437 (5)	
C1	0.37823 (19)	0.26508 (13)	0.44351 (16)	0.0339 (5)	
C2	0.4208 (2)	0.18520 (14)	0.41621 (18)	0.0410 (5)	
C3	0.3295 (2)	0.13620 (15)	0.3496 (2)	0.0486 (6)	
C4	0.2019 (3)	0.16596 (15)	0.3120 (2)	0.0499 (6)	
C5	0.1611 (2)	0.24676 (14)	0.34049 (18)	0.0412 (5)	
C6	0.25100 (19)	0.29646 (13)	0.40678 (16)	0.0344 (5)	
C7	0.24062 (19)	0.38483 (13)	0.45012 (16)	0.0332 (5)	
C8	0.37284 (19)	0.39541 (14)	0.52730 (16)	0.0347 (5)	
C9	0.59230 (19)	0.31952 (13)	0.55089 (17)	0.0368 (5)	
C10	0.6346 (2)	0.31418 (17)	0.66367 (19)	0.0522 (6)	
C11	0.7729 (3)	0.31052 (19)	0.6958 (2)	0.0633 (8)	
C12	0.8648 (3)	0.31018 (17)	0.6165 (2)	0.0564 (7)	
C13	0.8220 (2)	0.31587 (18)	0.5039 (2)	0.0588 (7)	
C14	0.6849 (2)	0.32088 (17)	0.4702 (2)	0.0512 (6)	
C15	0.2332 (2)	0.45561 (14)	0.36026 (17)	0.0361 (5)	
C16	0.1135 (2)	0.44411 (13)	0.27349 (18)	0.0383 (5)	
C17	−0.0177 (2)	0.45994 (15)	0.3006 (2)	0.0505 (6)	
C18	−0.1280 (3)	0.44404 (17)	0.2237 (3)	0.0631 (8)	
C19	−0.1082 (3)	0.41453 (19)	0.1173 (3)	0.0696 (8)	
C20	0.0215 (3)	0.39940 (19)	0.0871 (2)	0.0664 (8)	
C21	0.1298 (2)	0.41352 (15)	0.16628 (19)	0.0490 (6)	
H1	0.2197 (19)	0.5066 (14)	0.3987 (17)	0.037 (5)*	
H2	0.651 (2)	0.3249 (15)	0.393 (2)	0.060 (7)*	
H3	0.071 (2)	0.2662 (13)	0.3161 (16)	0.040 (6)*	
H4	0.508 (2)	0.1655 (13)	0.4453 (16)	0.040 (6)*	
H5	0.357 (2)	0.0802 (15)	0.3293 (18)	0.049 (6)*	
H6	0.803 (3)	0.3077 (17)	0.774 (2)	0.078 (8)*	
H8	−0.217 (3)	0.4545 (17)	0.244 (2)	0.080 (9)*	
H9	0.137 (2)	0.1290 (16)	0.266 (2)	0.061 (7)*	
H10	0.887 (3)	0.3167 (17)	0.447 (2)	0.075 (8)*	
H11	0.962 (3)	0.3091 (16)	0.642 (2)	0.074 (8)*	
H12	0.427 (3)	0.4894 (16)	0.346 (2)	0.065 (8)*	
H13	−0.183 (3)	0.4066 (19)	0.062 (2)	0.090 (9)*	
H14	−0.032 (2)	0.4815 (16)	0.374 (2)	0.066 (8)*	
H15	0.568 (2)	0.3155 (15)	0.716 (2)	0.062 (7)*	
H17	0.044 (3)	0.3776 (17)	0.015 (2)	0.078 (9)*	

### Atomic displacement parameters (Å^2^)

**Table:**

	*U*^11^	*U*^22^	*U*^33^	*U*^12^	*U*^13^	*U*^23^
S1	0.0534 (5)	0.0792 (9)	0.0403 (4)	−0.0260 (5)	0.0085 (3)	−0.0133 (5)
O2	0.0526 (13)	0.0712 (17)	0.0521 (12)	−0.0169 (13)	0.0062 (10)	−0.0275 (12)
O4	0.0994 (17)	0.0848 (17)	0.0546 (12)	−0.0396 (13)	−0.0075 (11)	0.0232 (11)
O3	0.0565 (12)	0.148 (2)	0.0774 (13)	−0.0243 (13)	0.0253 (10)	−0.0428 (14)
S1'	0.090 (8)	0.065 (12)	0.087 (10)	−0.045 (9)	0.042 (8)	−0.014 (8)
O2'	0.08 (2)	0.09 (3)	0.052 (17)	0.03 (2)	−0.006 (14)	0.014 (18)
O4'	0.11 (3)	0.046 (18)	0.08 (2)	−0.015 (16)	0.025 (17)	0.000 (14)
F1	0.0324 (7)	0.0573 (8)	0.0462 (7)	0.0019 (6)	0.0095 (5)	−0.0056 (6)
O1	0.0440 (9)	0.0443 (9)	0.0449 (8)	−0.0035 (7)	−0.0032 (7)	−0.0136 (7)
N1	0.0284 (9)	0.0409 (10)	0.0387 (9)	0.0014 (8)	−0.0017 (7)	−0.0056 (8)
N2	0.0416 (11)	0.0496 (12)	0.0398 (10)	−0.0153 (9)	0.0023 (8)	−0.0045 (9)
C1	0.0319 (10)	0.0379 (12)	0.0319 (10)	−0.0037 (9)	0.0027 (8)	−0.0028 (9)
C2	0.0370 (12)	0.0399 (13)	0.0458 (12)	0.0033 (10)	0.0023 (9)	−0.0013 (10)
C3	0.0546 (15)	0.0343 (13)	0.0560 (14)	0.0024 (11)	−0.0001 (11)	−0.0080 (11)
C4	0.0523 (15)	0.0365 (13)	0.0585 (14)	−0.0045 (11)	−0.0094 (11)	−0.0091 (11)
C5	0.0358 (12)	0.0388 (13)	0.0474 (12)	−0.0027 (10)	−0.0061 (10)	−0.0038 (10)
C6	0.0310 (10)	0.0358 (12)	0.0363 (10)	−0.0006 (9)	0.0016 (8)	0.0006 (9)
C7	0.0274 (10)	0.0368 (12)	0.0356 (10)	−0.0001 (8)	0.0039 (8)	−0.0063 (9)
C8	0.0315 (11)	0.0425 (13)	0.0302 (10)	−0.0035 (9)	0.0027 (8)	−0.0034 (9)
C9	0.0285 (10)	0.0408 (13)	0.0405 (11)	−0.0007 (9)	−0.0012 (8)	−0.0002 (10)
C10	0.0428 (13)	0.0735 (18)	0.0399 (12)	0.0008 (12)	−0.0002 (10)	0.0076 (12)
C11	0.0528 (16)	0.083 (2)	0.0511 (15)	0.0011 (14)	−0.0157 (13)	0.0125 (15)
C12	0.0349 (13)	0.0544 (16)	0.0777 (18)	0.0001 (11)	−0.0087 (12)	0.0057 (13)
C13	0.0360 (13)	0.0709 (19)	0.0704 (17)	−0.0016 (12)	0.0094 (12)	0.0001 (15)
C14	0.0384 (13)	0.0725 (18)	0.0426 (13)	−0.0039 (12)	0.0025 (10)	−0.0009 (12)
C15	0.0377 (12)	0.0318 (12)	0.0384 (11)	−0.0001 (9)	0.0005 (9)	−0.0067 (9)
C16	0.0398 (12)	0.0289 (12)	0.0446 (12)	−0.0013 (9)	−0.0058 (9)	0.0012 (9)
C17	0.0475 (14)	0.0420 (14)	0.0605 (16)	0.0092 (11)	−0.0047 (12)	0.0008 (12)
C18	0.0441 (15)	0.0549 (17)	0.087 (2)	0.0062 (13)	−0.0149 (14)	0.0103 (15)
C19	0.0598 (19)	0.0663 (19)	0.077 (2)	−0.0114 (15)	−0.0303 (16)	0.0128 (16)
C20	0.072 (2)	0.072 (2)	0.0518 (15)	−0.0189 (16)	−0.0151 (14)	−0.0037 (14)
C21	0.0480 (14)	0.0524 (15)	0.0458 (13)	−0.0107 (11)	−0.0023 (10)	−0.0028 (11)

### Geometric parameters (Å, º)

**Table:**

S1—O3	1.399 (2)	C6—C7	1.494 (3)
S1—O4	1.428 (3)	C7—C8	1.540 (3)
S1—O2	1.584 (2)	C7—C15	1.541 (3)
S1—N2	1.6086 (19)	C9—C10	1.368 (3)
O2—C21	1.417 (3)	C9—C14	1.375 (3)
O3—S1'	1.346 (14)	C10—C11	1.389 (3)
S1'—O4'	1.29 (4)	C10—H15	0.94 (2)
S1'—O2'	1.62 (4)	C11—C12	1.358 (4)
S1'—N2	1.636 (16)	C11—H6	0.95 (3)
O2'—C21	1.50 (4)	C12—C13	1.367 (4)
F1—C7	1.396 (2)	C12—H11	0.98 (3)
O1—C8	1.217 (2)	C13—C14	1.382 (3)
N1—C8	1.357 (3)	C13—H10	0.97 (3)
N1—C1	1.427 (2)	C14—H2	0.95 (2)
N1—C9	1.434 (2)	C15—C16	1.513 (3)
N2—C15	1.471 (3)	C15—H1	0.94 (2)
N2—H12	0.89 (3)	C16—C21	1.380 (3)
C1—C2	1.377 (3)	C16—C17	1.382 (3)
C1—C6	1.387 (3)	C17—C18	1.383 (3)
C2—C3	1.385 (3)	C17—H14	0.96 (3)
C2—H4	0.95 (2)	C18—C19	1.372 (4)
C3—C4	1.383 (3)	C18—H8	0.94 (3)
C3—H5	0.96 (2)	C19—C20	1.378 (4)
C4—C5	1.389 (3)	C19—H13	0.96 (3)
C4—H9	0.99 (2)	C20—C21	1.382 (3)
C5—C6	1.381 (3)	C20—H17	0.97 (3)
C5—H3	0.96 (2)		
			
O3—S1—O4	117.89 (16)	O1—C8—C7	124.78 (18)
O3—S1—O2	108.28 (17)	N1—C8—C7	107.73 (16)
O4—S1—O2	108.17 (16)	C10—C9—C14	120.7 (2)
O3—S1—N2	108.81 (13)	C10—C9—N1	120.65 (18)
O4—S1—N2	112.62 (14)	C14—C9—N1	118.59 (18)
O2—S1—N2	99.39 (13)	C9—C10—C11	119.0 (2)
C21—O2—S1	114.28 (18)	C9—C10—H15	117.9 (15)
O4'—S1'—O3	105 (2)	C11—C10—H15	123.1 (15)
O4'—S1'—O2'	114 (3)	C12—C11—C10	120.6 (2)
O3—S1'—O2'	115 (2)	C12—C11—H6	119.8 (16)
O4'—S1'—N2	115.1 (18)	C10—C11—H6	119.6 (16)
O3—S1'—N2	110.0 (11)	C11—C12—C13	120.2 (2)
O2'—S1'—N2	98.2 (19)	C11—C12—H11	118.4 (15)
C21—O2'—S1'	104 (2)	C13—C12—H11	121.3 (15)
C8—N1—C1	110.56 (16)	C12—C13—C14	120.2 (2)
C8—N1—C9	124.62 (17)	C12—C13—H10	121.0 (16)
C1—N1—C9	124.05 (16)	C14—C13—H10	118.9 (16)
C15—N2—S1	121.86 (15)	C9—C14—C13	119.3 (2)
C15—N2—S1'	119.7 (6)	C9—C14—H2	117.8 (14)
C15—N2—H12	114.1 (17)	C13—C14—H2	122.8 (14)
S1—N2—H12	110.6 (17)	N2—C15—C16	113.32 (17)
S1'—N2—H12	125.5 (18)	N2—C15—C7	106.41 (16)
C2—C1—C6	122.69 (19)	C16—C15—C7	111.82 (17)
C2—C1—N1	127.34 (18)	N2—C15—H1	111.1 (12)
C6—C1—N1	109.93 (17)	C16—C15—H1	107.3 (12)
C1—C2—C3	116.7 (2)	C7—C15—H1	106.8 (12)
C1—C2—H4	119.9 (12)	C21—C16—C17	117.3 (2)
C3—C2—H4	123.3 (12)	C21—C16—C15	121.5 (2)
C4—C3—C2	121.7 (2)	C17—C16—C15	121.1 (2)
C4—C3—H5	120.3 (13)	C16—C17—C18	121.1 (3)
C2—C3—H5	117.9 (13)	C16—C17—H14	119.1 (15)
C3—C4—C5	120.6 (2)	C18—C17—H14	119.8 (15)
C3—C4—H9	120.2 (14)	C19—C18—C17	120.1 (3)
C5—C4—H9	119.2 (14)	C19—C18—H8	120.0 (18)
C6—C5—C4	118.4 (2)	C17—C18—H8	119.9 (18)
C6—C5—H3	121.6 (12)	C18—C19—C20	120.2 (3)
C4—C5—H3	120.0 (12)	C18—C19—H13	120.7 (18)
C5—C6—C1	119.89 (19)	C20—C19—H13	119.0 (18)
C5—C6—C7	131.90 (19)	C19—C20—C21	118.7 (3)
C1—C6—C7	108.20 (17)	C19—C20—H17	125.3 (16)
F1—C7—C6	112.54 (15)	C21—C20—H17	116.0 (16)
F1—C7—C8	108.70 (14)	C16—C21—C20	122.6 (2)
C6—C7—C8	103.09 (16)	C16—C21—O2	119.2 (2)
F1—C7—C15	107.28 (15)	C20—C21—O2	118.1 (2)
C6—C7—C15	116.26 (16)	C16—C21—O2'	111.3 (15)
C8—C7—C15	108.69 (16)	C20—C21—O2'	113.9 (12)
O1—C8—N1	127.38 (18)		
			
O3—S1—O2—C21	174.48 (19)	C1—N1—C9—C10	−119.0 (2)
O4—S1—O2—C21	−56.7 (2)	C8—N1—C9—C14	−106.6 (2)
N2—S1—O2—C21	61.0 (3)	C1—N1—C9—C14	62.4 (3)
O4'—S1'—O2'—C21	51 (3)	C14—C9—C10—C11	0.3 (4)
O3—S1'—O2'—C21	172.2 (14)	N1—C9—C10—C11	−178.3 (2)
N2—S1'—O2'—C21	−71 (3)	C9—C10—C11—C12	−1.6 (4)
O3—S1—N2—C15	−157.38 (18)	C10—C11—C12—C13	1.9 (4)
O4—S1—N2—C15	70.0 (2)	C11—C12—C13—C14	−0.9 (4)
O2—S1—N2—C15	−44.3 (2)	C10—C9—C14—C13	0.7 (4)
O4'—S1'—N2—C15	−70 (2)	N1—C9—C14—C13	179.3 (2)
O3—S1'—N2—C15	171.6 (11)	C12—C13—C14—C9	−0.4 (4)
O2'—S1'—N2—C15	51 (2)	S1—N2—C15—C16	9.7 (3)
C8—N1—C1—C2	−176.2 (2)	S1'—N2—C15—C16	−23.9 (11)
C9—N1—C1—C2	13.4 (3)	S1—N2—C15—C7	132.95 (18)
C8—N1—C1—C6	1.3 (2)	S1'—N2—C15—C7	99.4 (11)
C9—N1—C1—C6	−169.02 (17)	F1—C7—C15—N2	167.10 (15)
C6—C1—C2—C3	−0.2 (3)	C6—C7—C15—N2	−66.0 (2)
N1—C1—C2—C3	177.03 (19)	C8—C7—C15—N2	49.7 (2)
C1—C2—C3—C4	0.1 (3)	F1—C7—C15—C16	−68.7 (2)
C2—C3—C4—C5	0.0 (4)	C6—C7—C15—C16	58.3 (2)
C3—C4—C5—C6	0.1 (3)	C8—C7—C15—C16	173.96 (16)
C4—C5—C6—C1	−0.2 (3)	N2—C15—C16—C21	16.9 (3)
C4—C5—C6—C7	178.8 (2)	C7—C15—C16—C21	−103.4 (2)
C2—C1—C6—C5	0.3 (3)	N2—C15—C16—C17	−166.4 (2)
N1—C1—C6—C5	−177.37 (18)	C7—C15—C16—C17	73.3 (3)
C2—C1—C6—C7	−178.93 (18)	C21—C16—C17—C18	1.3 (4)
N1—C1—C6—C7	3.4 (2)	C15—C16—C17—C18	−175.6 (2)
C5—C6—C7—F1	57.8 (3)	C16—C17—C18—C19	−2.1 (4)
C1—C6—C7—F1	−123.05 (17)	C17—C18—C19—C20	1.0 (4)
C5—C6—C7—C8	174.7 (2)	C18—C19—C20—C21	0.8 (4)
C1—C6—C7—C8	−6.1 (2)	C17—C16—C21—C20	0.6 (4)
C5—C6—C7—C15	−66.5 (3)	C15—C16—C21—C20	177.4 (2)
C1—C6—C7—C15	112.66 (19)	C17—C16—C21—O2	−176.3 (2)
C1—N1—C8—O1	178.27 (19)	C15—C16—C21—O2	0.5 (3)
C9—N1—C8—O1	−11.5 (3)	C17—C16—C21—O2'	140.5 (14)
C1—N1—C8—C7	−5.3 (2)	C15—C16—C21—O2'	−42.6 (14)
C9—N1—C8—C7	165.00 (16)	C19—C20—C21—C16	−1.6 (4)
F1—C7—C8—O1	−56.9 (2)	C19—C20—C21—O2	175.3 (3)
C6—C7—C8—O1	−176.50 (18)	C19—C20—C21—O2'	−140.6 (17)
C15—C7—C8—O1	59.6 (2)	S1—O2—C21—C16	−44.5 (3)
F1—C7—C8—N1	126.54 (17)	S1—O2—C21—C20	138.4 (2)
C6—C7—C8—N1	6.9 (2)	S1'—O2'—C21—C16	73 (2)
C15—C7—C8—N1	−117.00 (18)	S1'—O2'—C21—C20	−143.7 (16)
C8—N1—C9—C10	72.0 (3)		

### Hydrogen-bond geometry (Å, º)

**Table:**

*D*—H···*A*	*D*—H	H···*A*	*D*···*A*	*D*—H···*A*
N2—H12···O1^i^	0.89 (3)	1.99 (3)	2.868 (2)	166 (2)

Symmetry code: (i) −*x*+1, −*y*+1, −*z*+1.
